# Surgery for brain metastases: radiooncology scores predict survival-score index for radiosurgery, graded prognostic assessment, recursive partitioning analysis

**DOI:** 10.1007/s00701-022-05356-x

**Published:** 2022-09-24

**Authors:** Christina Wolfert, Veit Rohde, Abdelhalim Hussein, Ingo Fiss, Silvia Hernández-Durán, Dörthe Malzahn, Annalen Bleckmann, Dorothee Mielke, Bawarjan Schatlo

**Affiliations:** 1 Department of Neurosurgery, University Hospital Göttingen, Robert-Koch-Str. 40, 37075 Göttingen, Germany; 2 mzBiostatistics, Statistical Consultancy, 37075 Göttingen, Germany; 3 Clinic for Hematology/ Medical Oncology, University Medical Center Göttingen, Robert-Koch-Str. 40, 37075 Göttingen, Germany; 4 Medical Clinic A, Haematology, Haemostasiology, Oncology and Pulmonology, University Hospital Münster, 48149 Münster, Germany

**Keywords:** Cerebral metastasis, Scores, Survival, Neurooncology, Surgery for brain tumors

## Abstract

**Background:**

Radiooncological scores are used to stratify patients for radiation therapy. We assessed their ability to predict overall survival (OS) in patients undergoing surgery for metastatic brain disease.

**Methods:**

We performed a post-hoc single-center analysis of 175 patients, prospectively enrolled in the MetastaSys study data. Score index of radiosurgery (SIR), graded prognostic assessment (GPA), and recursive partitioning analysis (RPA) were assessed. All scores consider age, systemic disease, and performance status prior to surgery. Furthermore, GPA and SIR include the number of intracranial lesions while SIR additionally requires metastatic lesion volume. Predictive values for case fatality at 1 year after surgery were compared among scoring systems.

**Results:**

All scores produced accurate reflections on OS after surgery (*p* ≤ 0.003). Median survival was 21–24 weeks in patients scored in the unfavorable cohorts, respectively. In cohorts with favorable scores, median survival ranged from 42 to 60 weeks. Favorable SIR was associated with a hazard ratio (*HR*) of 0.44 [0.29, 0.66] for death within 1 year. For GPA, the *HR* amounted to 0.44 [0.25, 0.75], while RPA had a *HR* of 0.30 [0.14, 0.63]. Overall test performance was highest for the SIR.

**Conclusions:**

All scores proved useful in predicting OS. Considering our data, we recommend using the SIR for preoperative prognostic evaluation and counseling.

## Introduction

Brain metastases are more frequent than primary brain tumors [12, 16]. They may arise even years after primary diagnosis and worsen prognosis [12, 24]. A plethora of attempts have been undertaken to predict overall survival (OS) in this patient population. Among these factors are gender, the number of brain lesions, and functional status [6, 15, 18–20, 29]. The choice for or against surgery in patients with brain metastases is a recurring problem in neurooncological practice. However, three scores are well established for purposes of prognostic stratification in the realm of radiooncology: The score index for radiosurgery (SIR) is a five-item score validated to predict OS [10, 27]. The graded prognostic assessment (GPA) is based on four parameters and was developed with a similar aim [14]. Finally, the recursive partitioning analysis (RPA) generates three classes based on three items and is another easily applicable score and reliable of predicting OS [4, 13]. The purpose of the present study was to evaluate the validity of these scores and assess whether they could be aid clinicians in their decision-making process for resection of brain metastasis. A guideline on which score to use would be helpful. For this reason, the overall survival in patients prior to surgical treatment of metastatic brain disease was assessed.

## Methods

### Inclusion criteria

We performed a monocentric analysis of patients, prospectively enrolled in the MetastaSys trial, who underwent microsurgical resection of at least one brain metastasis at the Göttingen University Hospital, Germany, from June 2013 to December 2016. Indication for resection was based on current guidelines and interdisciplinary tumor board conferences. The resection of a metastatic brain tumor was indicated in (1) space occupying lesions with pronounced perifocal edema, (2) occlusive hydrocephalus due to the metastatic brain tumor, (3) singular or solitary metastases, and (4) progressive neurologic deterioration based on the space occupying effect. This study was performed in accordance with the Ethics committee of the Georg-August University, Göttingen (approval no. 24/10/05) and with the 1964 Helsinki Declaration and its amendments.

Patients were included in the present analysis if (1) they were older than 18 years of age at the time of surgery, (2) preoperative cranial MR imaging (T1 sequences with contrast media) showed at least one metastatic lesion and medical indication for resection of one or more lesions was given. Exclusion criteria were (1) patient unable or unwilling to consent by themselves/an authorized legal representative, (2) incomplete data, and (3) no surgical treatment or incomplete tumor removal. For this study, survival was evaluated at regular neuro-oncological follow-up visits and recorded in weeks after surgery. In cases of unavailable follow-ups, patients were contacted by telephone.

### Baseline characteristics

Baseline characteristics such as age, sex, and Karnofsky performance scale (KPS) were recorded prior surgery (Table 1). Details of the malignancy were assessed and evaluated for the origin of the primary tumor, the number of brain metastases, and the extracerebral disease status. Furthermore, location of the resected metastatic lesion was dichotomized into eloquent-precentral or postcentral gyrus, calcarine fissure, left frontal operculum, left inferior parietal lobule, left superior temporal gyrus (posterior part), dentate gyrus, internal capsule, basal ganglia, thalamus, hypothalamus- and non-eloquent brain regions as well as in supra- and infratentorial origin of the operated lesion.

**Table 1:** Patient and tumor characteristics

Characteristics	Mean	SD
Age (years)	63.5	10
Preoperative KPS	82.1	16.5
Volume of the operated lesion (cm^3^)	27.1	49.9
Number of cerebral metastases	3.1	3.8
	**N**	**%**
Male	95	54.3
Solitary cerebral lesion	86	49.1
Disease status		
Progressive	153	87.5
Stable disease	9	5.1
No extracerebral evidence of disease	13	7.4
Infratentorial lesion	73	41.7
Eloquent localization	46	26.3
Primary tumor origin		
Lung	83	47.4
Breast	26	14.9
Gastrointestinal tract	25	14.3
Melanoma	25	14.3
Kidney	5	2.9
Other	11	6.3
Postoperative treatment		
Radiation	151	86.3
Chemotherapy	115	65.7
Combined radio-chemotherapy	110	62.9

Systemic disease burden was assessed by interdisciplinary consensus based on computed tomography (CT) scans of the thorax and abdomen with iodinated contrast medium.

### Radiooncological scores

The scoring systems were evaluated based on the baseline characteristics and the already established categories were reviewed. These were, after confirmation of reliability, adopted unchanged for further analysis. In addition, possible extensions to the scores were calculated.

### Score index for radiosurgery (SIR)

As established by Lorenzoni et al. [10], five factors contribute to the SIR:

- Age (0 points: > 60; 1 point: 51–59; 2 points: < 50)
- KPS (0 points: < 50; 1 point: 60–70; 2 points: > 70)
- Systemic disease status (0 points: progressive disease; 1 point: partial remission, stable disease; 2 points: complete clinical remission, no evidence of disease)
- Largest lesion volume in cm.^3^ (0 points: > 13; 1 point: 5–13; 2 points: < 5)
- Number of cerebral lesions (0 points: > 3; 1 point: 2; 2 points: 1)

The sum of the aforementioned categories reflects the SIR which is subdivided into four classes (class 1: 0–3 points; class 2: 4 points; class 3: 5–7 points, class 4: 8–10 points). Patients in class 4 are expected to have favorable, while a minimum of points and classification in class 1 likely reflects an unfavorable baseline [10].

### Graded prognostic assessment (GPA)

Contrary to the SIR, the graded prognostic assessment (GPA) does not take the volume of the largest cerebral lesion into account. The GPA is calculated as follows [14, 22]:

- Age (0 points: > 60; 0.5 points: 50–60; 1 point: < 50)
- KPS (0 points: < 70; 0.5 points 70–80, 1 point: 90–100)
- Extracranial metastases (0 points: present; 1 point: absent)
- Number of brain metastases (0 points: > 3, 0.5 points: 2–3; 1 point: 1)

In 2010, the GPA has been updated with refinements to create a diagnosis-specific GPA (dsGPA), which solely contains significant factors for the examined primary tumor. Both, the GPA and the dsGPA are validated scores to assess the overall survival of patients suffering from metastatic brain disease. Due to the heterogeneity of the primary tumors in our patient cohort and thus the comparatively small number of patients with the same entity of primary tumors, the GPA was calculated for all patients included in our study.

The sum of the GPA scores was used to categorize patients (class 1: 0–1 point; class 2: 1.5–2 points; class 3: 2.5–3 points, class 4: 3.5–4 points). Higher GPA values reflect a more favorable baseline [14, 22].

### Recursive partitioning analysis (RPA)

In comparison with the categories used to build the SIR and the GPA, KPS is the crucial factor when applying the RPA. Patients are graduated into three classes as follows [10, 27]]:

- Class 1: Patient with KPS ≥ 70, and controlled primary tumor, and age < 65 years
- Class 3: Patients with KPS < 70
- Class 2: Patients not matching any of the other classes

Class 1 includes young patients with good functional status and controlled tumor disease, while class 3 encompasses patients with low KPS. Thus, a lower RPA category reflects a more favorable baseline status [10, 27].

### Statistical methods

Data are presented using means and standard deviations (SD) where appropriate. For categorical data, prevalences using percentages are given. Overall survival was analyzed with Cox-proportional hazards models using R software version 4.0.1 (R Core Team, Vienna, Austria). Reported *p*-values are two-sided and, where considered statistically significant, at *p* ≤ 0.05/3 when testing the SIR, GPA, and RPA or at *p* ≤ 0.05/7 when testing seven additional putative predictors combined with the SIR.

## Results

### Baseline characteristics

Out of the 175 patients, 95 (54.3%) were male with a mean age of 63.5 ± 10 years. In most cases (*n* = 102; 58.3%), the operated lesion was supratentorial, while infratentorial lesions were less common (*n* = 73; 41.7%). Eighty-six patients (49.1%) had a solitary brain lesion, whereas the remaining 89 (50.9%) suffered from a mean of 3.1 ± 3.8 lesions. Lung cancer was the predominant primary (*n* = 83; 47.4%), with non small-cell lung cancer in 63 patients (36.0%) and small cell lung cancer in the remaining 20 (11.4%; Table 1).

### Score index for radiosurgery (SIR)

In the summary, 59 patients (33.7%) could be classified in class 1, 49 (28.0%) in class 2, 62 (35.4%) in class 3, and 5 (2.8%) in class 4. Details are shown in Table 2.

**Table 2:** Data of the prognostic scores

SIR			
	0 points	1 point	2 points
Age (*n* / %)	117 / 66.9	35 / 20.0	23 / 13.1
KPS (*n* / %)	12 / 6.9	44 / 22.9	123 / 70.3
Systemic disease status (*n* / %)	148 / 84.6	14 / 8.0	13 / 7.4
Largest lesion volume in cm^3^ (*n* / %)	87 / 49.7	45 / 25.7	43 / 24.6
Number of cerebral lesions (*n*/%)	59 / 33.7	30 / 17.1	86 / 49.1
GPA			
	0 points	0.5 points	1 point
Age (*n* / %)	20 / 11.4	38 / 21.7	117 / 66.9
KPS (*n* / %)	26 / 14.9	65 / 37.1	84 / 48.0
Extracranial metastases (*n* / %)	13 / 7.4	-	162 / 92.6
Number of brain metastases (*n* / %)	43 / 24.6	46 / 26.3	86 / 49.1
RPA			
	Class 1	Class 2	Class 3
(*n* / %)	14 / 8.0	136 / 77.7	25 / 14.4

### Graded prognostic assessment (GPA)

The GPA was calculated according to the elaborated categories with 61 patients (34.9%) in class 1, 85 (48.6%) in class 2, 25 (14.3%) in class 3, and 4 (2.3%) in class 4 (Table 2).

### Recursive partitioning analysis (RPA)

Analyzing the RPA, 14 patients (8.0%) were classified in class 1, 25 (14.3%) class 3, and the remaining 136 patients (77.7%) did not match any of the aforementioned classes and were therefore categorized as class 2 (Table 2).

### Survival outcome

**Fig. 1:**
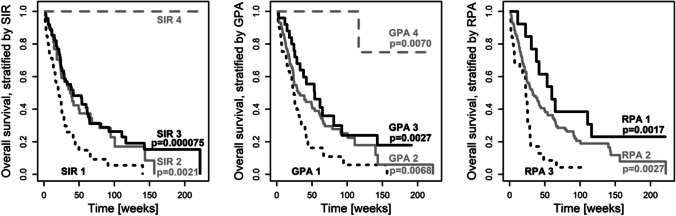
Preoperative SIR, GPA, and RPA predict long-term survival after surgical resection of brain metastasis. Overall survival in a surgical cohort (*n* = 175), stratified by the SIR (left), GPA (middle) and RPA (right). Within scoring systems, pairwise group comparisons (*p*-values) were obtained with respect to the group with worst outcome (black dotted lines: SIR class 1, GPA class 1, RPA class 3). See Table 2 for details

**Fig. 2:**
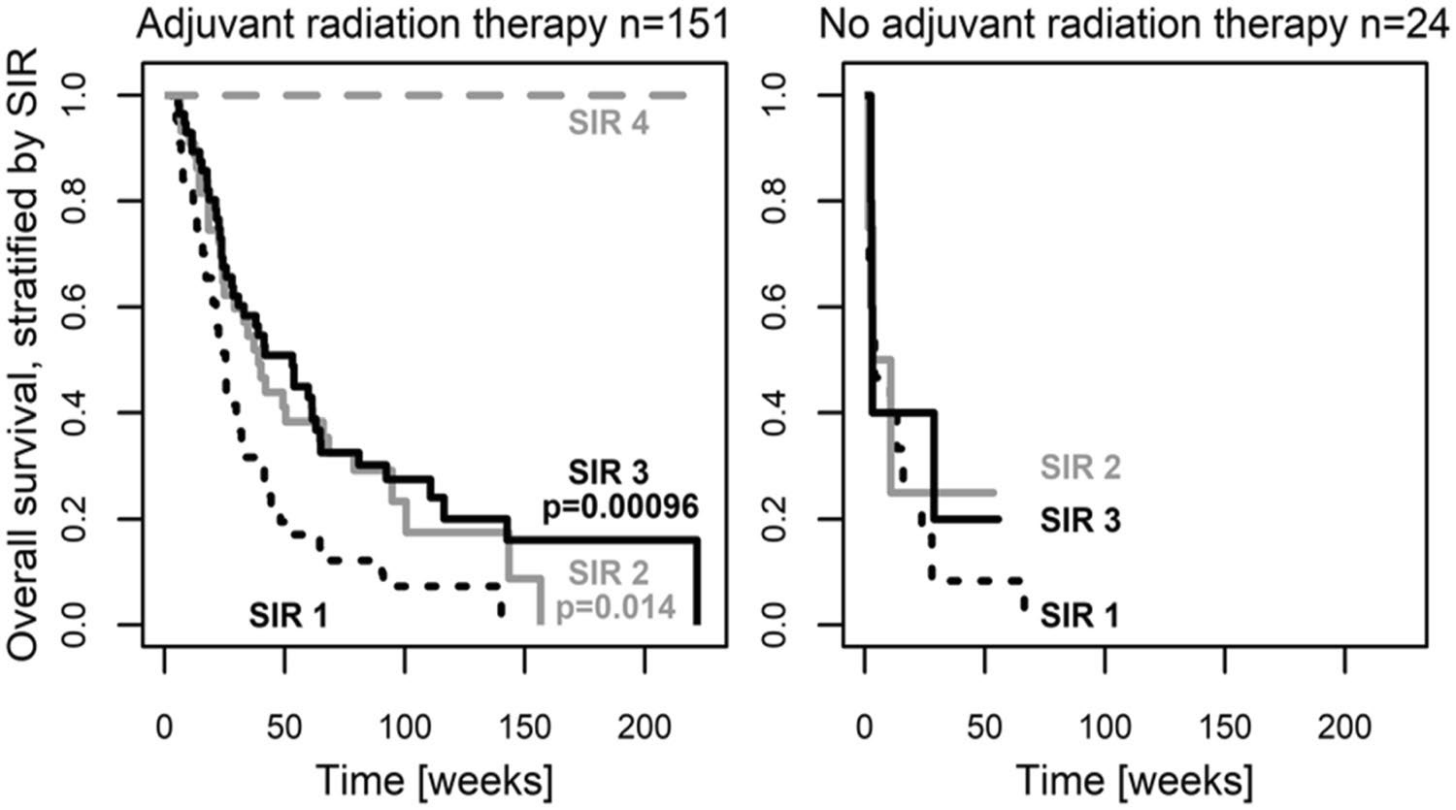
Postoperative radiotherapy and the SIR. The SIR distinguished long-term survival outcome especially in patients with postoperative radiotheray (left panel: 86% of the cohort, *n* = 151: 44 with SIR-1, 45 with SIR-2, 57 with SIR-3, 5 with SIR-4). Only few patients had received no postoperative radiotherapy (right panel: 14%, *n* = 24: 15 with SIR-1, 4 with SIR-2, 5 with SIR-3, none with SIR-4). For details, see Table 3

At the time of analysis, 135 (77.1%) were dead. Overall survival differed significantly between classes of the SIR (*p* = 0.00000005), the GPA (*p* = 0.00003), and the RPA (*p* = 0.003, global test over all classes, respectively). Within scoring systems, Fig. 1 and Table 3 display pairwise comparisons with SIR class 1, GPA class 1, and RPA class 3 who had a median survival of 21 to 24 weeks (Fig. 1 black dotted line). Median survival was prolonged to 42 to 60 weeks (Fig. 1 black solid line) in SIR class 3 (*HR* [95% *CI*]: 0.44 [0.29, 0.66]), GPA class 3 (*HR* [95% *CI*]: 0.44 [0.25, 0.75]), and RPA class 1 (*HR* [95% *CI*]: 0.30 [0.14, 0.63], *p* < 0.003). Among scoring systems, the best model of overall survival was provided by the SIR as having the highest likelihood of correct prediction (*p* < 0.000001) with the established classes of the SIR (*p* = 0.016). Complementing the SIR with additional predictive factors was not supported for infratentorial origin of the resected lesion (*p* = 0.88), eloquent location (*p* = 0.84), patient gender (*p* = 0.29), surgical indication for resection (*p* = 0.29), postoperative chemotherapy (*p* = 0.20), nor for origin of the primary tumor (*p* = 0.055). However, it was beneficial to combine the SIR with the binary factor adjuvant radiation therapy (*p* = 0.00023). Adjuvant radiation therapy, administered to 151 patients (86%), prolonged survival (*HR* [95% *CI*]: 0.36 [0.22, 0.59], *p* = 0.000048, adjusted for the SIR). Especially the improved survival in patients who received adjuvant radiotherapy (Fig. 2 left versus right panel) did depend on the SIR (Table 4).

**Table 3:** Overall survival differed between classes of the SIR, GPA, and RPA

Score	Class	*n* (events)	Median survival [95% *CI*] in weeks	*HR* [95% *CI*]	*p*-value^a^
SIR	1	59 (54)	21 [16, 28]	Reference	**–**
	2	49 (35)	37 [24, 68]	0.51 [0.33, 0.78]	**0.0021**
	3	62 (46)	42 [29, 65]	0.44 [0.29, 0.66]	**0.000075**
	4	5 (0)	NA [NA, NA]	**–**	**–**
GPA	1	61 (54)	24 [20, 32]	Reference	**–**
	2	85 (62)	31 [24, 60]	0.60 [0.42, 0.87]	**0.0068**
	3	25 (18)	54 [33, 143]	0.44 [0.25, 0.75]	**0.0027**
	4	4 (1)	NA [116, NA]	0.06 [0.009, 0.47]	**0.0070**
RPA	3	25 (23)	23 [15, 29]	Reference	**–**
	2	136 (102)	33 [24, 49]	0.49 [0.31, 0.78]	**0.0027**
	1	14 (10)	60 [38, NA]	0.30 [0.14, 0.63]	**0.0017**

*CI*, confidence interval; *GPA*, graded prognostic assessment; *HR*, hazard ratio; *n*, sample size; *NA*, no value; *RPA*, recursive partitioning analysis; *SIR*, score index for radiosurgery

^a^Univariate analysis of overall survival with respect to the SIR, the GPA, and the RPA

**Table 4:** Postoperative radiotherapy and the SIR are important predictors

Predictor		*n* (events)	Median survival [95% *CI*], in weeks	*HR* [95% *CI*]	*p*-value
Postoperative radiotherapy	No	24 (21)	3.6 [2.9, 24]	Reference	**–**
	Yes	151 (114)	38 [29, 49]	0.36 [0.22, 0.59]	**0.000048**^**a**^
SIR, in *n* = 151 patients with postoperative radiotherapy	SIR 1	44 (40)	25 [20, 32]	Reference	**–**
	SIR 2	45 (32)	39 [25, 79]	0.56 [0.35, 0.89]	**0.014**^**b**^
	SIR 3	57 (42)	53 [31, 65]	0.47 [0.30, 0.74]	**0.00096**^**b**^
	SIR 4	5 (0)	NA [NA, NA]	**–**	**–**

*CI*, confidence interval; *HR*, hazard ratio; *NA*, no value; *SIR*, score index for radiosurgery

^a^Multivariate analysis: Inferential statistics (*HR*, 95% *CI*, *p*-value) of the effect of postoperative radiotherapy are adjusted for the SIR (*n* = 175, whole cohort)

^b^Univariate analysis of the SIR in the stratum of patients with postoperative radiotherapy (*n* = 151, 86% of the cohort)

## Conclusion

### Key findings

All three radiooncological scores SIR, GPA, and RPA provided useful categories to predict long-term outcome prior to surgical resection of brain metastases. The four classes of the SIR were statistically most solid in setting apart survival of greater than 1 year after surgery. Compared to the GPA and RPA, the SIR encompassed more information and yielded more precise estimates of survival and more homogenously distributed sample sizes across classes.

### Other studies

Resection of metastatic brain lesions allows histopathological analysis of tumor tissue, which helps in identifying the origin of the primary tumor. This is essential in patients with unknown primaries. Furthermore, neurological deficits resulting out of mass effect, caused by the lesion itself or surrounding edema, frequently require surgical removal of the brain metastasis [21].

In patients with glioma, the extent of resection is correlated with improved survival, whereas the impact of resection on long-term survival in patients with metastatic brain lesions remains a matter of debate [7, 11, 20]. Regarding metastasis treatment, the only level 1 evidence for resection is proven in patients harboring a single metastatic lesion, measuring 3 to 4 cm in diameter, but adequate data providing the survival benefit in patients with multiple cerebral lesions is scarce [9, 25]. Current guidelines solely provide low-level evidence recommendations, and significant heterogeneity exists in treatment strategies among centers [2].

In a recent multicenter study, an external validation model was developed to predict functional impairment after intracranial tumor surgery [23]. Including 2437 patients, this model portrayed the importance of lesion location within the brain, surgical approach, tumor histology, sex, and KPS at admission [23]. The aim of the present study was to assess if presently available tools, e.g., radiooncological scores, can be translated into surgical decision-making. By virtue of having only three distinct groups, the RPA is the most intuitive and least cumbersome score, which in our hands, was also useful in predicting long-term survival. Gaspar et al. had previously confirmed its solidity in a large dataset of more than a thousand patients who underwent radiation therapy, albeit without surgery [4]. Agboola et al. confirmed this finding in patients after surgical resection and concomitant radiotherapy [1]. Golden et al. on the other hand criticized that the primary tumor site is another significant variable to be factored into prognostic considerations [5]. The GPA is more intricate than the RPA and was modified into the ds-GPA which is now in parallel use with the original GPA [22]. To facilitate the comparability of score, we focused on the original GPA in our current study.

With five parameters, the SIR is the most complex of the studied rating systems and was praised for its strong predictive power [27]. Patient age itself, touted as an important risk factor for postoperative morbidity and mortality, increases several risk factors that lead to an increased morbidity and mortality [26]. Furthermore, the functional status is a decisive factor for long-term survival. The recent study of Staartjes et al. confirmed significance of KPS at admission to predict further neurological impairment after resection in intracranial lesions [23]. Low KPS goes hand in hand with cerebral and systemic disease burden which, in an intertwined manner, translates into poor OS [3]. For this reason, the systemic disease burden, showing significant impact on overall survival in every metastatic disease, is the third category of the SIR [8]. Finally, the intracerebral lesions are analyzed according to their multiplicity and volume. While level I evidence for resection is proven in patients with singular lesions, level IIIb evidence for resection is given in patients with a good KPS and controlled systemic disease status harboring 2 or 3 metastatic brain lesions [2, 25]. In these patients, results after complete surgical resection are comparable to those obtained in patients with single lesions [17].

Comparing the results of our prospectively enrolled patient cohort with the retrospective analysis of the Norwegian Brain Tumor Registry from Winther et al., the same tumor entities are included [28]. However, the primary tumor entities are slightly differently represented with an elevated number of lung and breast cancer in our cohort (47.4% vs 33%; 14.9% vs. 9%). The Norwegian study examines the GPA as well as the DS-GPA, however, due to the limited number of patients and the hereby small sample size evaluation of the Ds-GPA, did not seem well applicable in our study. Nevertheless, the baseline data which is essential to form the scores are comparable to our cohort and to the already validated scores that were used in our study additionally.

The unadjusted regression analyses performed by Winther et al. identified male gender, increasing age, an elevated ECOG status, multiple brain metastases, the presence of comorbidities, and extracranial metastases as well as a progressive, synchronous, or unknown extracranial disease status to be associated with shorter OS.

The GPA is analyzed with age, KPS, the number of intracranial metastases, and the presence of extracranial lesions. All of these factors are mentioned above and are identified by Winther et al. separately to be associated with shorter OS. Furthermore, the RPA applied in our study is predominantly based on the KPS, including age and systemic disease status. In this score, the identified risk factors for shorter OS are represented, accordingly.

The most accurate score in our study, the SIR, subdivides the systemic disease status not only into present or absent metastases but also further into progressive disease, partial remission — stable disease and complete clinical remission. The number of brain metastases and patients age is subdivided accordingly to the GPA. The KPS is more strictly subdivided than in the GPA that above all, it is evident which patients are already suffering from a greater impairment preoperatively and therefore will not receive any further tumor therapy. The only category applied in the SIR and not evaluated by Winther and colleagues is the largest lesion volume. However, four out of five categories are identified in the regression analysis by Winther et al. in their analysis of 590 patients with comparable survival rates. Hereby, a cross-validation with their study cohort seems applicable in our point of view and strengthens the expressive power of our study [28].

Regarding the results obtained in our analysis, the SIR, partitioned in its four established classes, was validated in patients with surgical resection of one or more brain metastases with/without consecutive radiotherapy. Tumor characteristics, such as eloquent location and infra-/supratentorial origin, were not influential enough to merit addition to the SIR. In contrast, postoperative radiation therapy was a strong additional predictor. However, this factor cannot be taken into account preoperatively since the decision on further tumor treatment can only be provided after but not prior surgery. Therefore, we advocate the preoperative use of the SIR to estimate postoperative long-term survival of patients with metastatic brain disease.

Based on the Metastasys study data, the SIR provides the best estimate of OS in patients prior to surgery for metastatic brain disease. The use of this score can be recommended to assist radiation oncologists and surgeons alike in the pre-therapeutic decision-making process.

## Data Availability

The data that support the findings of this study are not openly available due to reasons of sensitivity and are available from the corresponding author upon reasonable request.
